# Effects of salt stress on ion balance and nitrogen metabolism of old and young leaves in rice (*Oryza sativa* L.)

**DOI:** 10.1186/1471-2229-12-194

**Published:** 2012-10-21

**Authors:** Huan Wang, Meishan Zhang, Rui Guo, Decheng Shi, Bao Liu, Xiuyun Lin, Chunwu Yang

**Affiliations:** 1 Key laboratory of Molecular Epigenetics of MOE, Northeast Normal University, Changchun 130024 Jilin Province, China; 2 Department of Agronomy, Jilin Agricultural University, Changchun, 130118, Jilin Province, China; 3 Institute of Environment and Sustainable Development in Agriculture(IEDA), Chinese Academy of Agricultural Sciences (CAAS), Key Laboratory of Dry land Agriculture, MOA, Beijing, 100081, China; 4 Rice Institute, Jilin Academy of Agricultural Sciences, Changchun, 130033, Jilin Province, China

**Keywords:** Salt stress, Rice, Nitrogen metabolism, Gene expression, Old and young leaves

## Abstract

**Background:**

It is well known that salt stress has different effects on old and young tissues. However, it remains largely unexplored whether old and young tissues have different regulatory mechanism during adaptation of plants to salt stress. The aim of this study was to investigate whether salt stress has different effects on the ion balance and nitrogen metabolism in the old and young leaves of rice, and to compare functions of both organs in rice salt tolerance.

**Results:**

Rice protected young leaves from ion harm via the large accumulation of Na^+^ and Cl^−^ in old leaves. The up-regulation of *OsHKT1;1*, *OsHAK10* and *OsHAK16* might contribute to accumulation of Na^+^ in old leaves under salt stress. In addition, lower expression of *OsHKT1;5* and *OsSOS1* in old leaves may decrease frequency of retrieving Na^+^ from old leaf cells. Under salt stress, old leaves showed higher concentration of NO_3_^−^ content than young leaves. Up-regulation of *OsNRT1;2*, a gene coding nitrate transporter, might contribute to the accumulation of NO_3_^−^ in the old leaves of salt stressed-rice. Salt stress clearly up-regulated the expression of *OsGDH2* and *OsGDH3* in old leaves, while strongly down-regulated expression of *OsGS2* and *OsFd-GOGAT* in old leaves.

**Conclusions:**

The down-regulation of *OsGS2* and *OsFd-GOGAT* in old leaves might be a harmful response to excesses of Na^+^ and Cl^−^. Under salt stress, rice might accumulate Na^+^ and Cl^−^ to toxic levels in old leaves. This might influence photorespiration process, reduce NH_4_^+^ production from photorespiration, and immediately down-regulate the expression of *OsGS2* and *OsFd-GOGAT* in old leaves of salt stressed rice. Excesses of Na^+^ and Cl^−^ also might change the pathway of NH_4_^+^ assimilation in old leaves of salt stressed rice plants, weaken GOGAT/GS pathway and elevate GDH pathway.

## Background

Salinity is one of most prevalent abiotic stresses that limit crop productivity in arid and semi-arid regions. Salt tolerance of plants is a complex phenomenon that involves morphological and developmental changes as well as physiological and biochemical processes [1,2]. Response by plants to salt stress is a complex network affecting almost all processes, including nutrient uptake and metabolism, ion accumulation and photosynthesis. Salt stress in the soil generally involves osmotic stress and ion injury [3]. High salt environments can break the ion homeostasis of plant cells, destroy the ionic balance, and affect the distributions of K^+^ and Na^+^ in the cells [4]. It is necessary to re-establish the ion homeostasis in cells for plant living under salt-stress [4]. Plant survival and growth in saline environments is a result of adaptive processes such as ion transport and compartmentation, compatible solutes synthesis and accumulation. Many of these compatible solutes are N-containing compounds, such as amino acids and betaines, hence the nitrogen metabolism is of central importance for salt tolerance [5].

It is well known that salt stress has different effects on old and young tissues [6]. For example, salt stress produces distinct effects on the growth and Na^+^ accumulation of old and young leaves [7-11]. It is widely believed that old and young tissues play distinct roles in salt tolerance and plants protect young leaves from ion harm via the large accumulation of toxic ions like Na^+^ in old leaves during adaptation to salt stress [3,7-12]. Thus, the understanding of comparative effects of salt stress on old and young tissues may be important for salt tolerance research. Although plant biologists have extensively reported the difference between old and young tissues, many questions remain. For example, how do old and young tissues regulate ion balance in response of plants to salt stress? whether salt stress has different effects on nitrogen metabolism of old and young leaves?

Salt stress in soil generally involves osmotic stress and ion-induced injury, and Na^+^ is the main toxic ion in salinized soil. Low Na^+^ and high K^+^ in the cytoplasm are essential for the maintenance of a number of enzymatic processes. Transmembrane K^+^ movements in plants are mediated by several types of channels, including the AKT family, and by transporters that belong to two families, KcsA-TRK (HKT) and KUP/HAK/KT (HAK). The extent of tolerance by plants to Na^+^ stress depends on ion compartmentalization and exclusion. In higher plants, the salt overly sensitive protein 1 (SOS1) functions in Na^+^ exclusion from root epidermal cells into the rhizosphere, which also plays a role in retrieving Na^+^ from shoots to roots [3]. SOS3–SOS2 (CIPK24-CBL4) protein kinase pathway mediates regulation of the expression and activities of Na^+^ transporters such as SOS1 and Na^+^/H^+^ exchanger (NHX) that mediates Na^+^ compartmentalization into vacuoles [3]. In rice plants, a high affinity K^+^ transporter (HKT) family, OsHKT1;5, mediates Na^+^ exclusion from shoots via Na^+^ removal from the xylem sap [3].

Plant roots absorb nitrate (NO_3_^–^), ammonium (NH_4_^+^), and other nutrients from soil using a variety of transporters. For example, AMT protein family members transport NH_4_^+^ and NRT protein family members transport nitrate. NO_3_^–^ is reduced to nitrite by nitrate reductase (NR) and then to NH_4_^+^ by nitrite reductase (NiR). NH_4_^+^ from both nitrate reduction and soil are incorporated into amino acid by glutamine synthetase (GS) and glutamate synthase (Fd-GOGAT and NADH-GOGAT) or alternative glutamate dehydrogenase (GDH) pathway [13].

The study was designed to investigate whether salt stress has different effects on the ion balance and nitrogen metabolism in the old and young leaves of rice, and to compare functions of both organs in rice salt tolerance. The rice seedlings were subjected to salt stress. The contents of inorganic ions, NH_4_^+^-nitrogen, and NO_3_^−^-nitrogen in old and young leaves were then measured. The expression of some critical genes involved in ion balance and nitrogen metabolism also were assayed to test their roles in salt tolerance.

## Results

### Ion accumulation

**Figure 1:**
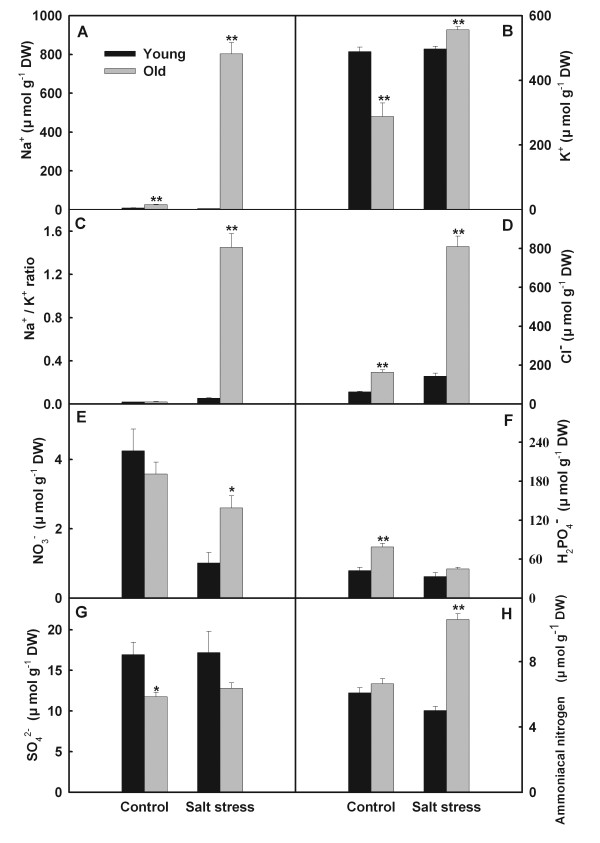
**Effects of salt stress on the contents of inorganic ions in young and old leaves of rice seedlings.** The values are means (± SE) of four biological replicates, and each replicate consisted of a pool of 10 plants. Statistically significant between organs at same stress condition was determined by *t*-test, and marked as * (*P* < 0.05) and ** (*P* < 0.01).

Salt stress only slightly influenced accumulations of Na^+^, K^+^, Na^+^/K^+^ ratio, Cl^–^ and ammoniacal nitrogen in young leaves, but strongly stimulated their accumulation in old leaves (Figure 1). Salt stress reduced NO_3_^−^ contents in both old and young leaves, with reduction in young leaves greater than in old leaves. Although the NO_3_^−^ content was much higher in old leaves than young leaves under salt stress (Figure 1E), substantial differences between both organs are not found in their SO_4_^2-^ and H_2_PO_4_^−^ contents (Figure 1).

### Ion metabolism

**Figure 2:**
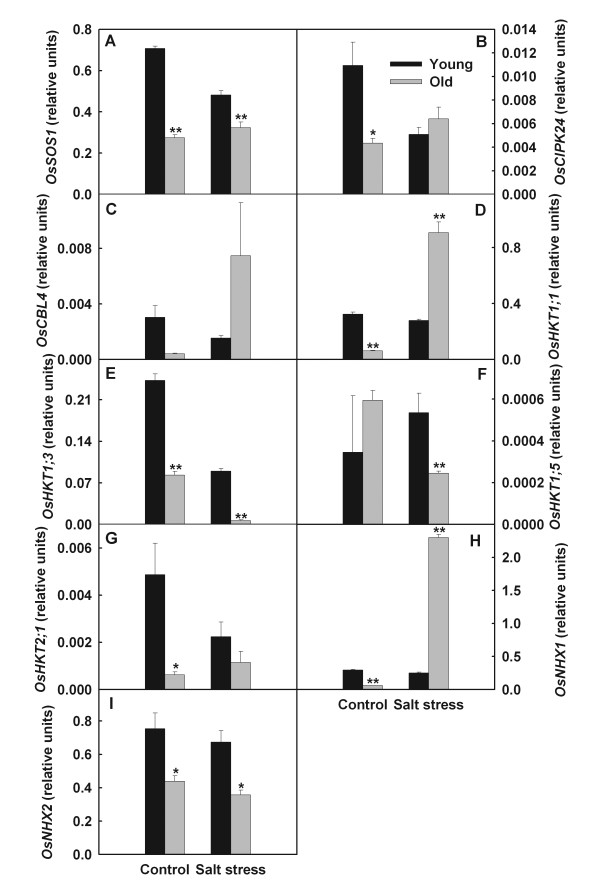
**Effects of salt stress on the expression of *OsSOS *pathway genes and *OsHKT *gene family in young and old leaves of rice seedlings.** The values are means (± SE) of four biological replicates, and each replicate consisted of a pool of 10 plants. Statistically significant between organs at same stress condition was determined by *t*-test, and marked as * (*P* < 0.05) and ** (*P* < 0.01).

**Figure 3:**
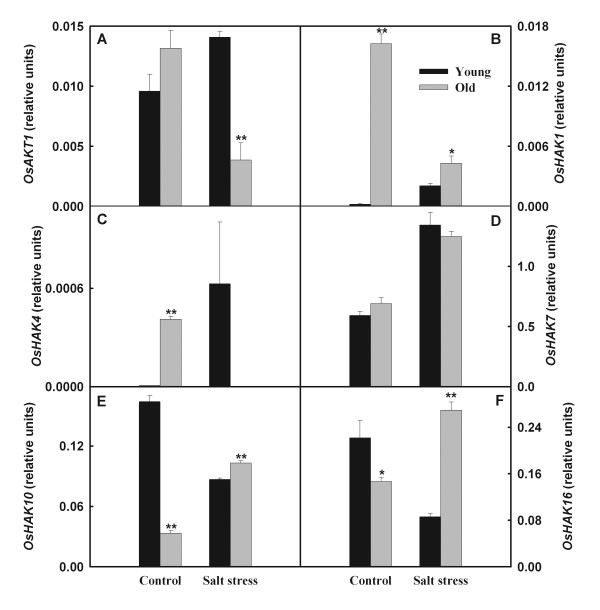
**Effects of salt stress on the expression of *OsAKT1 *and *OsHAK *gene family in young and old leaves of rice seedlings.** The values are means (± SE) of four biological replicates, and each replicate consisted of a pool of 10 plants. Statistically significant between organs at same stress condition was determined by *t*-test, and marked as * (*P* < 0.05) and ** (*P* < 0.01).

Salt stress slightly down-regulated the expression levels of *OsCBL4*, *OsHKT1;1* and *OsNHX1* in young leaves, while strongly stimulated their expression in old leaves (Figure 2). Salt stress stimulated the expression of *OsHKT1;5* in young leaves but down-regulated its expression in old leaves (Figure 2F). The expression of *OsHKT1;3* and *OsHKT2;1* in young leaves were lower than old leaves (Figure 2). Expression levels of *OsHAK10* and *OsHAK16* in young leaves were down-regulated by salt stress, whereas their expression levels in old leaves were enhanced (Figure 3). Salt stress mightily up-regulated expression of *OsHAK4* in young leaves but reduced its expression in old leaves (Figure 3C). The expression level of *OsAKT1* in old leaves clearly decreased, while in young leaves increased (Figure 3A).

### NH_4_^+^ assimilation

**Figure 4:**
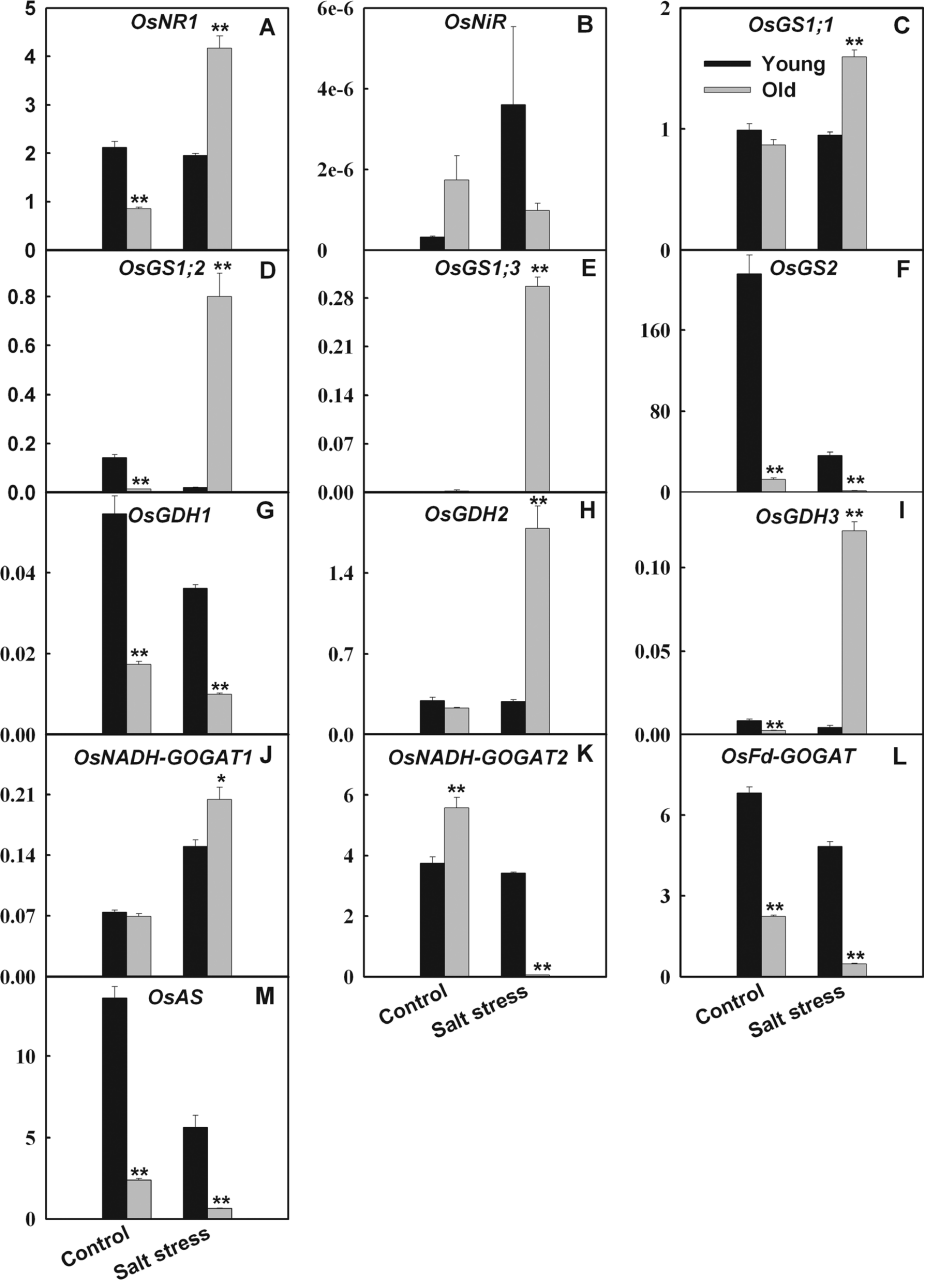
**Effects of salt stress on the expression (relative units) of genesinvolved in NH**_**4**_^**+**^**assimilation in young and old leaves of rice seedlings.** The values are means (± SE) of four biological replicates, and each replicate consisted of a pool of 10 plants. Statistically significant between organs at same stress condition was determined by *t*-test, and marked as * (*P* < 0.05) and ** (*P* < 0.01).

**Figure 5:**
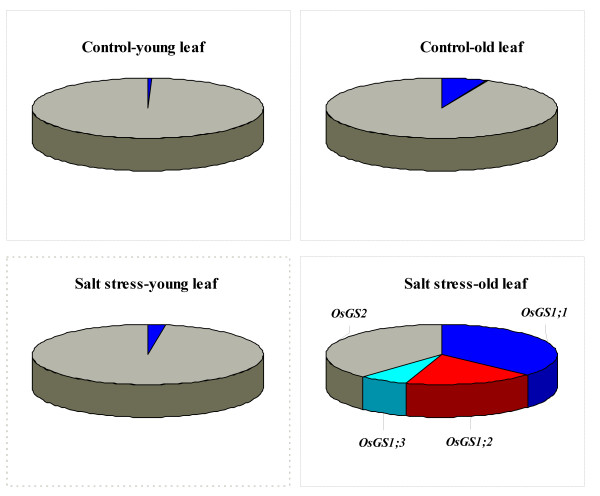
**Percent contribution of different *OsGS *gene family members to total *OsGS *expression in young and old leaves of rice seedlings under salt stress.**

Effects of salt stress on the expression of *OsNR1*, *OsGS1;1*, *OsGS1;2*, *OsGS1;3*, *OsGDH2* and *OsGDH3* in young leaves were insignificant, while their expression in old leaves were strongly up-regulated (Figure 4). Salt stress lowered the expression of *OsNiR* in old leaves, and enhanced its expression in young leaves. The expression levels of *OsNADH-GOGAT2*, *OsAS*, *OsGS2* and *OsFd-GOGAT* in both organs were down-regulated by salt stress (Figure 4)*,* while expression levels of the four genes all were much lower in old leaves than in young leaves. Under control condition, expression level of *OsGS2* was much higher than other *OsGS* family members (Figure 5). Under salt stress, percent contribution of *OsGS2* to total *OsGS* expression in young leaves still was much higher than other members; however, in old leaves, expression of all *OsGS*1 gene family members were clearly up-regulated and resulted in the reduction of percent contribution of *OsGS2* (Figure 5).

### Nitrogen uptake

**Figure 6:**
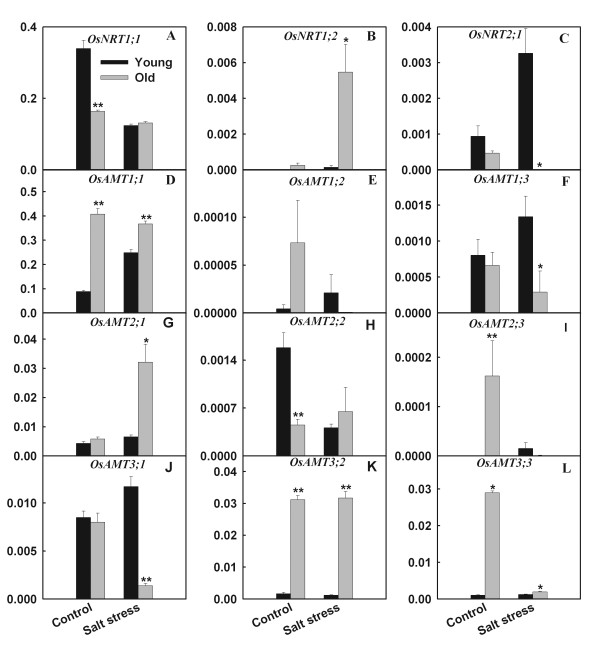
**Effects of salt stress on the expression (relative units) of *OsAMT *and *OsNRT *gene families in young and old leaves of rice seedlings.** The values are means (± SE) of four biological replicates, and each replicate consisted of a pool of 10 plants. Statistically significant between organs at same stress condition was determined by *t*-test, and marked as * (*P* < 0.05) and ** (*P* < 0.01).

Salt stress strongly stimulated the expression of O*sNRT1;2* in old leaves, but down-regulated the expression of *OsNRT2;1* in old leaves and up-regulated the expression of *OsNRT2;1* in young leaves (Figure 6). Expression level of *OsAMT1;1* was much higher than other *AMT* gene family members (Table 1). Responses of *OsAMT* family members to salt stress were diverse. For example, salt stress strongly enhanced the expression of *OsAMT1;1* in young leaves but up-regulated the expression of *OsAMT2;1* in old leaves (Figure 6). In addition, salt stress strongly reduced the expression of *OsAMT1;2*, *OsAMT2;3*, *OsAMT3;1* and *OsAMT3;3* in old leaves.

**Table 1:** **Percent contribution of different *OsAMT *gene family members to total *OsAMT *expression in young and old leaves of rice seddlings under salt stress**

	**Young leaf**		**Old leaf**	
	**Control**	**Salt stress**	**Control**	**Salt stress**
*OsAMT1;1(%)*	83.142	91.732	84.405	84.36
*OsAMT1;2 (%)*	0.004	0.008	0.015	0.0002
*OsAMT1;3 (%)*	0.757	0.494	0.137	0.067
*OsAMT2;1 (%)*	4.059	2.413	1.198	7.3793
*OsAMT2;2 (%)*	1.490	0.152	0.094	0.1488
*OsAMT2;3 (%)*	0.001	0.006	0.034	0.0003
*OsAMT3;1 (%)*	8.000	4.313	1.656	0.3169
*OsAMT3;2 (%)*	1.567	0.434	6.463	7.2836
*OsAMT3;3 (%)*	0.979	0.449	5.998	0.4434

## Discussion

### Ion balance

Salt stress has a more complex effect on the ion balance in rice old leaves than in young leaves. The results indicated that salt stress only slightly influenced the accumulations of Na^+^, K^+^, Cl^−^ and ammoniacal nitrogen in young leaves, but salt stress strongly stimulated their accumulation in old leaves (Figure 1). Interestingly, salt stress did not limit accumulations of SO_4_^2-^ and H_2_PO_4_^−^ in rice leaves. It might be main reason for this that salt stress did not influence uptake of SO_4_^2-^ and H_2_PO_4_^−^. Rice plants protected young leaves from ion injury via mightily accumulating toxicant ions in old leaves. Mature leaves have a larger vacuole while young leaf cells only have dispersed miniature vacuoles [13]. Rice plants may transport toxicant ions (e.g. Na^+^ and Cl^−^) into the vacuoles of old leaves to alleviate the ion toxicity of whole plant.

**Figure 7:**
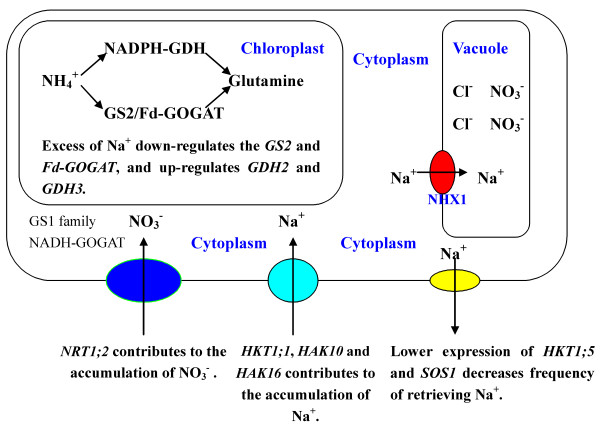
**Ion balance and nitrogen metabolism responses of old leaves of rice seedlings under salt stress.** Under salt stress, old leaves accumulates much higher concentrations of Na^+^, Cl^−^ and NO_3_^−^ than young leaves. Under salt stress, up-regulation of *OsHKT1;1*, *OsHAK10* and *OsHAK16* advances the accumulation of Na^+^ in old leaves, and increased *OsNHX1* expression contributes to Na^+^ compartmentalization in old leaves. In addition, lower expression of *OsHKT1;5* and *OsSOS1* in old leaves decreases frequency of retrieving Na^+^ from old leaf cells. Excess of Na^+^ weakens GOGAT/GS pathway and elevates GDH pathway in old leaves. The up-regulation of *OsNRT1;2*, a gene coding nitrate transporter, advances the accumulation of NO_3_^−^ in the old leaves of salt stressed-rice.

Under salt stress, Na^+^ competes with K^+^ for uptake into roots [14]. When Na^+^ enters cells and accumulates to high levels, it becomes toxic to enzymes. Tolerance of plants to Na^+^ stress relies on Na^+^ compartmentation at the cellular and tissue levels, Na^+^ exclusion and the control of long-distance transport in vasculatures [13]. Salt overly sensitive (SOS) salt tolerance pathway may play important roles in Na^+^ release from leaves to roots. It has been reported that rice OsHKT1;5 mediates Na^+^ release from shoots to roots [3,8]. In higher plants, the Na^+^/H^+^ exchanger (NHX) family mediates Na^+^ compartmentalization into vacuoles [6]. Under salt stress, up-regulated *OsNHX1* expression might contribute to Na^+^ compartmentalization in old leaves. Up-regulation of *OsHKT1;1*, *OsHAK10* and *OsHAK16* may advance the accumulation of Na^+^ in old leaves of salt stressed-rice (Figure 7). In addition, lower expression of *OsHKT1;5* and *OsSOS1* in old leaves may decrease frequency of retrieving Na^+^ from old leaf cells (Figure 7).

### Nitrogen metabolism

Nitrogen metabolism regulation is of central importance for salt tolerance, and interference between salinity and nitrogen nutrition is a very complex network affecting almost all processes [5]. Our results indicated that salt stress has a stronger effect on nitrogen metabolism in old leaves than in young leaves. Under salt stress, old leaves showed higher concentration of NO_3_^−^ content than young leaves. Under salt stress, these scarce NO_3_^−^ may be principally stored in old leaves with larger vacuole to keep the NO_3_^−^ supply in shoots [13]. The up-regulation of *OsNRT1;2*, a gene coding nitrate transporter, might advance the accumulation of NO_3_^−^ in the old leaves of salt stressed-rice. Our results also showed that salt stress has different effects on *OsAMT* gene family expression of young and old leaves (Figure 6). This indicated that young and old leaves might have different NH_4_^+^ transmission mechanisms under salt stress.

It is well known that NO_3_^–^ is reduced to nitrite by nitrate reductase (NR) and then to NH_4_^+^ by nitrite reductase (NiR). NH_4_^+^ is incorporated into amino acid by glutamine synthetase (GS) and glutamate synthase (Fd-GOGAT and NADH-GOGAT) or alternative glutamate dehydrogenase (GDH) pathway (reviewed by Shi et al. 2010) [15]. Glutamine synthetase (GS) includes two isozymes: GS1 in the cytosol and GS2 in chloroplasts/plastids [16]. *OsNADH-GOGAT* is mainly expressed in roots and mediates assimilation of NH_4_^+^ in roots, and Fd-GOGAT exists in chloroplast and functions in assimilation of NH_4_^+^ from photorespiration [13]. Our results indicated that the expression levels of *OsFd-GOGAT* and *OsGS2* in old leaves were much lower than those in young leaves in response of rice plants to salt stress. The down-regulation of *OsGS2* and *OsFd-GOGAT* in old leaves might be a harmful response to excesses of Na^+^ and Cl^−^. Under salt stress, rice might accumulate Na^+^ and Cl^−^ to toxic levels in old leaves (Figure 1A). This might harm the chloroplast, influence photorespiration process, and reduce NH_4_^+^ production from photorespiration, which immediately down-regulates the *OsGS2* and *OsFd-GOGAT* in old leaves of salt stressed rice (Figure 7). Excesses of Na^+^ and Cl^−^ even changed the pathway of NH_4_^+^ assimilation in old leaves, weakened GOGAT/GS pathway and elevated GDH pathway (Figure 7). We found that salt stress clearly elevated the expression levels of *OsGDH2* and *OsGDH3* in old leaves, while strongly down-regulated the expression of *OsGS2* and *OsFd-GOGAT* in old leaves (Figure 4). In addition, salt stress could change spatial distribution of various members of same gene family at whole plant level. For example, *OsGS1;3* is present only in the spikelets [16], but salt stress strongly stimulated its expression in old leaves (Figure 4E). Under control condition, expression level of *OsGS2* was much higher than other *OsGS* family members (Figure 5). Under salt stress, percent contribution of *OsGS2* to total *OsGS* expression in young leaves still was much higher than other members; however, in old leaves, expression of all *OsGS*1 gene family members were clearly up-regulated and resulted in the reduction of percent contribution of *OsGS2* (Figures 5 and 6).

## Conclusion**s**

Salt stress has a more complex effect on the ion balance in rice old leaves than in young leaves. Rice plants protect young leaves from ion injury via mightily accumulating toxicant ions in old leaves. Mature leaves have a larger vacuole while young leaf cells only have dispersed miniature vacuoles. Rice plants may transport toxicant ions (e.g. Na^+^ and Cl^−^) into the vacuoles of old leaves to alleviate the ion toxicity of whole plant. Under salt stress, up-regulation of *OsHKT1;1*, *OsHAK10* and *OsHAK16* advances the accumulation of Na^+^ in old leaves. In addition, lower expression of *OsHKT1;5* and *OsSOS1* in old leaves decreases frequency of retrieving Na^+^ from old leaf cells. Excess of Na^+^ weakens GOGAT/GS pathway and elevates GDH pathway in old leaves***.*** The up-regulation of *OsNRT1;2* advances the accumulation of NO_3_^−^ in the old leaves of salt stressed-rice.

## Methods

### Plant growth conditions

Matsumae, a major rice cultivar in north China, was chosen as the test organism. Matsumae as salt tolerance rice cultivar can grow in the moderate-salinized field of northeast China. Seeds were germinated and grown in petri dishes for 6 d in a growth cabinet (29°C during the day and 25°C during the night, 16/8 h photoperiod at 250 μmol m^–2^ s^–1^). Seedlings were then transferred to buckets containing 2000 mL of sterile nutrient solution for solution culture. The nutrient solution was replaced daily. The buckets were placed in a growth chamber that was maintained at 27.0 ± 1.5°C during the day and 22.0 ± 1.5°C during the night, under a 16/8 h photoperiod at 250 μmol m^–2^ s^–1^. The nutrient solution used in this work accorded to the components described by the International Rice Research Institute [17], and contained 1.44 mM NH_4_NO_3_, 0.32 mM NaH_2_PO_4_, 0.6 mM K_2_SO_4_, 1.0 mM CaCl_2_, 1.6 mM MgSO_4_, 0.072 mM Fe-EDTA, 0.2 mM Na_2_SiO_3_, 9.1 μM MnCl_2_, 0.154 μM ZnSO_4_, 0.156 μM CuSO_4_, 18.5 μM H_3_BO_3_ and 0.526 μM H_2_MoO_4_ at pH 5.2.

### Stress treatment

After 22 days of growth in hydroponic medium, rice plants were subjected to salt stress (100 mM NaCl) by transferring them to another barrel containing 2000 mL of the treatment solution amended with the above nutrients and 100 mM NaCl. A bucket including 20 seedlings represented one replicate, and there were four replicates per treatment. 8 buckets of seedlings were randomly divided into 2 sets, four buckets per set. Each bucket was considered as one replicate with four replicates per set, one set was used as control, and another set was treated with salt stress. Treatment solutions were replaced daily. The nutrient solution without stress salts was used as control. The 20 seedlings in each bucket were harvested after treatment for 6 d.

### Measurements of physiological indices

Old leaf was defined as second leaf at bottom, and young leaf as newly emerged leaf. The young and old leaves of 10 seedlings in each bucket were separated and mixed, then immediately frozen in liquid nitrogen and then stored at −70°C for RNA isolation. Another 10 seedlings in each bucket were washed with distilled water, after which the old and young leaves were separated and freeze-dried. Dry samples of plant material were treated with 10 mL deionized water at 100°C for 1 h, and the extract used to determine the contents of free inorganic ions. The contents of NO_3_^–^, Cl^–^, H_2_PO_4_^–^, and SO_4_^2–^ were determined by ion chromatography (DX-300 ion chromatographic system; AS4A-SC ion-exchange column, CD M-II electrical conductivity detector, mobile phase: Na_2_CO_3_/NaHCO_3_ = 1.7/1.8 mM; DIONEX, Sunnyvale, USA). Ammoniacal nitrogen was measured by ninhydrin colourimetry methods [18]. A flame photometer was used to determine K^+^ and Na^+^ contents.

### Quantitative real time PCR analysis

We extracted the total RNA from the young and old leaves of seedlings grown under stress or non-stress conditions using TRIzol reagent (Invitrogen). The RNA was treated with DNaseI (Invitrogen), reverse-transcribed using SuperScriptTM RNase H-Reverse Transcriptase (Invitrogen), and then subjected to real-time PCR analysis using gene-specific primers. The gene-specific primers and corresponding references are listed in Additional file 1. PCR amplification was conducted with an initial step at 95°C for 1 min followed by 45 cycles of 5 s at 95°C, 10 s at 60°C and 30 s at 72°C. Amplification of the target gene was monitored every cycle by SYBR Green. Amplification of the rice *UBQ5* (GenBank Accession AK061988) mRNA was used as an internal quantitative control [19-21]. The relative expression of the target genes was calculated using the △Ct method [22]. We optimized PCR reaction system, after which the amplification efficiencies of each target gene and reference gene were approximately equal.

### Statistical analysis

Statistical analysis of the data was performed using the statistical program SPSS 13.0 (SPSS, Chicago, USA). All data were represented by an average of the four biological replicates and the standard errors (S.E.). Statistically significant between old and young leaves at same stress condition was determined by *t* test.

## Abbreviations

NR: Nitrate reductase; NiR: Nitrite reductase; GOGAT: Glutamate synthase; GS: Glutamine synthetase; GDH: Glutamate dehydrogenase; AS: Asparagine synthetase; NRT: Nitrate transporter; AMT: Ammonium transporter; NHX: Na^+^/H^+^ exchanger; HKT: High affinity K^+^ transporter; HAK: KUP/HAK/KT K^+^ transporter; AKT: Low affinity K^+^ transporter; SOS: Salt overly sensitive; CBL: Calcineurin B-like protein; CIPK: CBL-interacting protein kinase.

## Competing interests

The authors have declared that no competing interests exist.

## Authors' contributions

CY and HW designed the study. CY, HW, and RG performed the experiments. CY, HW, XL, BL and DS analysed the data. CY and HW wrote the manuscript, which was further edited by BL and DS. All authors read and approved the final manuscript.

## Supplementary Material

Additional file 1 **Table S1. **Gene-specific primers used in real time PCR analysis. Click here for file
