# Supplementary material for: Effects of salt stress on ion balance and nitrogen metabolism of old and young leaves in rice (Oryza sativa L.)
Source: BMC Plant Biol. 2012 Oct 21;12:194. doi: 10.1186/1471-2229-12-194 (PMC3496643; doi:10.1186/1471-2229-12-194)
Supplement: Additional file 1 — Table S1. Gene-specific primers used in real time PCR analysis. [file 1471-2229-12-194-S1.doc]

Table S1: Gene-specific primers used in real time PCR analysis.

| **Gene name** | **GenBank Accession No.** | **Forward primer (5’-3’)** | **Reverse primer (5’-3’)** |
| --- | --- | --- | --- |
| *OsNHX1* | AB021878 | GTTCAAGAGTTACAACAAAGCACG | CAGCGGGAATACAAAAGCAG |
| *OsNHX2* | AY360145 | ACCAAGACGAAACACCCCTAC | AACCCAGCAACTACTCCAAGAA |
| *OsHKT1;1* | AJ491816 | ATTAGCAGAGCACTGTGGAGGAA | CCGACGAACCCGTAGGAAG |
| *OsHKT1;3* | *AJ491818* | *CAGTTCATCTACCAAAACAATCCA* | *AATACCTCACCACCAATCAGCA* |
| *OsHKT1;5* | DQ148410 | TGCCACCTTACACCACTTTCG | TGCCATACGCACTGATAACCTC |
| *OsHKT2;1* | AB061311 | GCATATTCACCCATTCTGGATTCAGT | CGATGGTGATGAGGCTGGAAAGT |
| *OsSOS1* | AY785147 | CTCCGTGCTCATAGAATCGC | ATACTCACTCAAGTGGGTCAATACC |
| *OsCBL4* | AK101368 | GGCATCGTTCGGATTTCAC | GAGATTCGCCTTTCTGCTGTT |
| *OsCIPK24* | AK102270 | AAGAAGCGGGTGGGGAGGT | GCGGTGGTTGAGGATGGTGT |
| *OsAKT1* | AY065970 | TACGACCGCCGATACAGAA | CCAAATAAGCCACAAAGAAGG |
| *OsHAK1* | AK119883 | TGGCGTTCCAGAGCGTG | GGGGATGATGATGAGGGTGTA |
| *OsHAK4* | AF129485 | CGTTCCCATCCGTCAGTAAA | CAGCCTCTGGTCTGGTTCGTC |
| *OsHAK7* | AJ427971 | GAACTCCAACTTCCTCAAGACG | AGATCATGCCGACTTCGACGAG |
| *OsHAK10* | AJ427972 | CGCTCTCGGCTGCTTTCCT | TAACCGCCAATCCTGACGC |
| *OsHAK16* | AJ427973 | AGCGACTGTGTGCTAAACCC | CATAGATGCCAATCCCTGAGA |
| *OsNR1* | AK121810 | CCTACTACTAAATTATACGCACCG | CAGGAAGGAATCAACCGCTA |
| *OsNiR* | AK103604 | CAAATCAGTGTTCCGATAGGTAA | GGCTGGAGACGGTGGTG |
| *OsGS1;1* | AB037595 | CCGTCTGTCGGCATTTCTG | GGGATGGGCTTGGGGTC |
| *OsGS1;2* | AB180688 | GCCCACAGGGACCATACTACT | CGTTGATGCCACTGATGTTGAT |
| *OsGS1;3* | AB180689 | CCGATTCCGACGAACAACC | GCTCCCGCCGCACAGT |
| *OsGS2* | X14246 | AGTATGCGTGAAGATGGAGGAT | GCCCCACCCGAATAGAGC |
| *OsNADH-GOGAT1* | AB008845 | TTGCGGTTACAAGACACTCTACTG | GCTCCCGTCCCTCCATCA |
| *OsNADH-GOGAT2* | AB274818 | CCTGTCGAAGGATCGTGAAGGTCAAACC | TGCATGGCCCTACTGTCTTCGCATCA |
| *OsFd-GOGAT* | AJ132280 | TGGTCTCCGCCCAGCAC | CAGTTTGTAGGTCAACCGTTATCAT |
| *OsGDH1* | AK071839 | TTCTTCCTTCCCACTACCAAAC | TCCCAAGCAGCGAGCC |
| *OsGDH2* | AB189166 | GGCCATTAACAACACTCATA | ACGCCGATCTATCTTGAAT |
| *OsGDH3* | AB035927 | TATGCTACTGAGGCTTTATTGACTG | GCCACCTTTCTGATGGATGA |
| *OsAS* | D83378 | GCCCTATTTACCTAAGCACATTC | AGGCTGCGTCCCATTCA |
| *OsNRT1;1* | AF140606 | GGGCAGAGTTCAGCAATCG | GGAAGGACGCCGCAGGT |
| *OsNRT1;2* | AY305030 | GCGGCGAGTCCCTGAG | CGACGGCGTAGATGAATGA |
| *OsNRT2;1* | AK072215 | ACGGCACAAAGTACAAGACG | CCACTGCGGGAAGTAGATG |
| *OsAMT1;1* | AK073718 | GCCTCCAACAGCAACAACC | CCAAACAGAAACTGGCAATCA |
| *OsAMT1;2* | NM_001053990 | CACGGTGGCGATGAAAGG | TTGGAGATGGTGGTGAAGGAC |
| *OsAMT1;3* | AK107204 | TCAAGCAGGTCCCACAGG | TGAGGAAGGCGGAGTAGATG |
| *OsAMT2;1* | AB051864 | GATGAATCACGCCGAAACAC | GCACGGACGAATCGCTACTT |
| *OsAMT2;2* | AB083582 | CGACCAAGGACAGGGAGA | CACGGCGAGCGAGGAG |
| *OsAMT2;3* | AK102106 | GTTCACCCCGCTCTGGC | CCGCTCCCTGTCGCTCTT |
| *OsAMT3;1* | AK120352 | CCAACTGCTGAAAAGTGAAAACG | TGCTTCGCATACGGCTGAC |
| *OsAMT3;2* | AK069311 | CCCAGTTCGGCAAGCAG | TGGCGAGGCAGATGAGG |
| *OsAMT3;3* | AK108711 | GAGATTCCCGCCCAACAA | TCCACCCAAGCCACAGC |
| *OsUBQ5* | AK061988 | ACCACTTCGACCGCCACTACT | ACGCCTAAGCCTGCTGGTT |

NR, nitrate reductase; NiR, nitrite reductase；GOGAT, glutamate synthase; GS, glutamine synthetase; GDH, glutamate dehydrogenase; AS, asparagine synthetase. NRT, nitrate transporter; AMT, ammonium transporter; NHX, Na^+^/H^+^ exchanger; HKT, high affinity K^+^ transporter; HAK, KUP/HAK/KT K^+^ transporter; AKT, low affinity K^+^ transporter; SOS, salt overly sensitive; CBL, calcineurin B-like protein; CIPK, CBL-interacting protein kinase.
